# Electrolyte outpatient clinic at a local hospital – experience from diagnostics, treatment and follow-up

**DOI:** 10.1186/s12913-020-5022-0

**Published:** 2020-02-28

**Authors:** Kiarash Tazmini, Anette Hylen Ranhoff

**Affiliations:** 1 Department of Medicine, Diakonhjemmet Hospital, Oslo, Norway; 2 Department of Endocrinology, Morbid Obesity and Preventive Medicine, Faculty of Medicine, Oslo University Hospital, Postbox 4950 Nydalen, 0424 Oslo, Norway; 3 Department of Clinical Science, University of Bergen, Postboks 7804, 5020 Bergen, Norway

**Keywords:** Electrolytes, Hyponatremia, Outpatient clinic, Hospital readmissions, Quality of life

## Abstract

**Background:**

Electrolyte imbalances (EI) are common among patients. Many patients have repeated hospitalizations with the same EI without being investigated and treated. We established an electrolyte outpatient clinic (EOC) to diagnose and treat patients with EI to improve symptoms and increase their quality of life (QoL). In addition, we also wanted to reduce the number of admissions with the same EI.

**Methods:**

Uncontrolled before-after study reporting experiences from this outpatient clinic as a quality assurance project. From October 2010 to October 2015, doctors at our local hospital and general practitioners could refer adult patients with EI to the EOC. Ninety patients with EI were referred, of whom 60 were included. Medical history, clinical examination and laboratory tests were performed, and results registered. Admissions with the same EI were recorded 1 year before and 1 year after consultation at the EOC. Patients responded to a questionnaire, composed by the authors, about symptoms before the first consultation, as well as symptom and QoL improvement after the last consultation.

**Results:**

Hyponatremia was the reason for referral in 45/60 patients. The total number of admissions with the same EI 1 year before the first consultation was 71, compared with 20 admissions 1 year after the last consultation. Improvement of symptoms was reported by 60% of patients, and 62% reported improvement in QoL.

**Conclusions:**

An EOC may be an appropriate way to organize the assessment and treatment of patients with EI.

## Background

Electrolyte imbalances (EIs) are common and are associated with increased morbidity and mortality [1, 2]. The prevalence of hyponatremia is up to 44.0%, hypocalcemia 25.3%, hypomagnesemia 20.1%, hypophosphatemia 20.0%, hyperkalemia 13.0%, hypernatremia 4.4%, and hypercalcemia 3.0% among hospitalized patients [1, 3, 4]. EI can be caused by renal insufficiency and age changes in the neurohumoral mechanisms, but can also be caused by different medical conditions like cardiovascular disease, lung diseases and gastrointestinal diseases [1, 5]. Many medications like diuretics, beta-blockers, ACE-inhibitors, potassium-sparing diuretics, laxatives, anti-epileptics, cytostatics also lead to EIs [2, 5, 6].

Hyponatremia is associated with longer hospital stay, a 15% risk of 30-day readmissions and increased hospital costs. Except for some studies of hyponatremia and readmission [7–10], the frequency of readmissions among patients with other EIs is poorly studied. Especially, relatively little data are available regarding outcomes of dysphosphatemia, dysmagnesemia, and dyscalcemia.

Hyponatremia is shown as an independent predictor of intensive care stay [7, 9]. Even mild hyponatremia (133–136 mmol/L) has been shown to be independently associated with mortality, and the risk of death increases with the worsening of hyponatremia [11].

Symptoms of hyponatremia are nonspecific, especially when mild to moderate, and can easily be overlooked. Clinicians will often accept sodium values above 120 mmol/L, and patients are often considered as asymptomatic [12].

Despite these negative consequences of hyponatremia, the condition is often underdiagnosed and insufficiently treated. This is partly due to the inadequate requisition of laboratory tests such as urine electrolytes and urine osmolality [13, 14].

In the literature, relatively little is described about the importance of diagnosis and treatment of EIs and the use of hospital resources. Our experience is that many patients are repeatedly readmitted with the same EI without being adequately investigated and treated. To provide these patients with better and more systematic investigation and treatment, we started an electrolyte outpatient clinic (EOC) organized in a medical outpatient clinic. The purpose of this study was to evaluate whether such an outpatient clinic could contribute to diagnostics and treatment, improvement of symptoms and quality of life (QoL), and fewer admissions with EI.

## Methods

We report experiences from a quality assurance project designed as an uncontrolled before-and-after study. From October 2010 to October 2015, doctors at Diakonhjemmet Hospital and general practitioners in the hospital catchment area could refer adult patients with EIs to the EOC, which was a part of the medical outpatient clinic. Diakonhjemmet Hospital is a local urban hospital in Oslo, Norway, for approximately 135,000 inhabitants. We included patients ≥18 years with an EI that was not diagnosed by the referring physician, and who could be followed up at an outpatient clinic. These patients were included at the first consultation at the EOC. Patients who were diagnosed and had started treatment before referral were excluded. One physician (KT) working in the hospital where the study was held considered referrals, investigated, treated and followed up all patients. Patients were examined and investigated as indicated in Table 1 according to their EI.

**Table 1:** Investigation at first consultation

A thorough medical history was taken since electrolyte imbalances can cause nonspecific symptoms (such as nausea, headaches, drowsiness, etc.), in addition to dietary history and fluid intakes per day, as well as urination and defecation. A clinical examination was performed, including an orthostatic blood pressure test (to assess volume status), and a thorough drug history (including natural remedies) was performed, as many drugs may lead to electrolyte imbalances. Patients were weighed, and their respective heights were given.	
Patients with hyponatremia were asked about antidiuretic hormone (ADH) stimuli: pain, nausea, stress and anxiety.	
**General blood samples were taken for all patients:** hemoglobin, C-reactive protein, leukocytes, sodium, potassium, albumin, glucose, creatinine, urea and eGFR in addition to urine electrolytes (depending on the electrolyte imbalance).	
**The following additional tests were taken depending on the electrolyte imbalance**	
*Hyponatremia*	
Blood tests: effective osmolality, thyroid-stimulating hormone (TSH), free T4, adrenocorticotropic hormone (ACTH), cortisol, aldosterone, renin activity. None of the patients had hyperglycemia, thus there was no need for glucose correction of serum sodium.	
Urine tests: sodium, potassium, creatinine, uric acid and osmolality.	
*Hyperkalemia*	
Serum and plasma potassium in addition to blood gas and platelets.	
*Hypomagnesaemia*	
Blood tests: magnesium, phosphate, free calcium, TSH, free T4.	
Urine tests: magnesium and creatinine. Fractional excretion of magnesium (FEMg) was calculated.	
*Hypophosphatemia*	
Blood tests: phosphate, magnesium, free calcium, blood gas, parathyroid hormone (PTH), and 25-OH vitamin D if suspicion of vitamin D deficiency.	
Urine tests: phosphate and creatinine. Fractional excretion of phosphate (FEPO4) was calculated.	
*Calcium disturbances*	
Blood tests: free calcium, PTH, 25-OH vitamin D, and blood gas.	
Urine tests: calcium and creatinine. Urine calcium/urine creatinine ratio.	

### Data collection

Date of referral, first and last consultation, and the total number of consultations were obtained from the patient administration system. Patients’ demographics and clinical data; gender, age, body mass index, chronic diseases, and Charlson Comorbidity Index [15], who referred the patient, and the reason for referral were registered. Body mass index and Charlson comorbidity index were calculated based on findings at the first consultation. We recorded symptoms by history at the first consultation at the EOC, especially those that could be attributed to EI. Electrolyte values at referral, the first and last consultation were also registered. Diagnostics (the cause of the EI) and type of interventions (discontinuation of drug, new medication/change of dosage, fluid restriction, and advice and information) were recorded.

In patients who received multiple interventions, the last intervention was registered. Treatment with tolvaptan in patients with SIADH was predefined.

We recorded the number of admissions with the same EI based on the laboratory data, 1 year before the first consultation and 1 year after the last consultation. Readmission was defined as two or more admissions with the same EI. The “index” emergency department visit that prompted referral to the EOC was not included. The EI could be the primary disorder or a concomitant finding at admission/during hospitalization. Admissions without a current EI were not recorded. In addition, patients responded to a questionnaire (supplementary material), composed by the authors, about symptoms before the first consultation, as well as symptom and QoL improvement after the last consultation. The questionnaire was given to the patients after the last consultation and was filled out outside the hospital. The patients were also asked about their experiences and satisfaction with the practice of the EOC at the last consultation.

### Statistical analysis

Continuous variables (age, BMI, time from referral to first consultation, time from first to last consultation, total number of consultations, Charlson comorbidity index, serum sodium levels) were reported with median and interquartile range (IQR). Frequency and proportion were given for categorical variables (sex, comorbidity, intervention on comorbidity, and type of treatment measures). Changes in serum sodium from the first to the last consultation were tested for statistical significance using the Wilcoxon signed-rank test. Admissions with EIs were divided into three categories: none, one admission, or two or more admissions (readmissions). We used McNemar’s Chi-square test for difference in number of admissions for each of the three categories 1 year before and 1 year after the EOC. Data were analyzed in Stata/SE (version 14.2; Stata Corporation, College Station, TX, USA).

### Ethics

Written consent to collect and store personal and health information was obtained from the patients. The research committee at Diakonhjemmet Hospital and the data protection officer for research and quality assurance approved the project as a quality assurance project, reference number 2011/21373.

## Results

Ninety patients were referred of whom 23 were excluded. Nine patients did not show up or cancelled the appointment, in nine patients follow-up was not possible, and in five patients consultation was considered unnecessary based on the information in the referral letters. Of the 67 patients that were appropriate for inclusion, informed consent was obtained from 60. Patient characteristics are listed in Table 2. Of these 60 patients, 34 were referred by internists at the hospital, while 22 patients were referred by general practitioners and four by other hospital doctors.

**Table 2:** Patient characteristics (*N* = 60)

	Median (interquartile range)
Age (*n* = 60)	69 (63–81) years
- Female (*n* = 42)	71 (63–82) years
- Male (*n* = 18)	68 (63–71) years
	**Proportion**
Sex (*n* = 60)	
- Female (*n* = 42)	42/60
- Male (*n* = 18)	18/60
	**Median (interquartile range)**
BMI (*n* = 58)	22.1 (19.2–24.2)
- Female (*n* = 40)	20.7 (19.1–23.6)
- Male (*n* = 18)	24.0 (21.0–25.4)
	**Median (interquartile range)**
Time from referral to 1st consultation (*n* = 60)	29 (21.5–43.5) days
Time from first to last consultation (*n* = 60)	57.5 (0–134) days
Total number of consultations (*n* = 60)	2 (1–3)
	**Proportion**
Comorbidity (*n* = 60)	57/60
- Hypertension	29/60
- Hypothyroidism	17/60
- Chronic kidney disease (eGFR < 60)	9/60
- Cerebrovascular disease	7/60
- Osteoporosis	7/60
- Arthritis	5/60
- Coronary artery disease	5/60
- Diabetes mellitus	4/60
- Chronic obstructive pulmonary disease	4/60
- Anxiety/depression	3/60
- Atrial fibrillation	3/60
- Cancer	3/60
Intervention on comorbidity	26/60
	**Median (interquartile range)**
Charlson comorbidity index^a^ (*n* = 60)	3 (2–4)

^a^ Charlson Comorbidity Index predicts one-year mortality for a patient with several comorbidities. An index of three gives an estimated 10-year survival rate of 77%

Hyponatremia was the reason for referral in 45/60 patients, while 15/60 had other EDs (hypernatremia, hyperkalemia, hypomagnesemia, hypophosphatemia, hypo-, and hypercalcemia). The 15 patients with other EIs than hyponatremia are not described further because of patient privacy.

### Hyponatremia

Among the 45 patients with hyponatremia, 27 were diagnosed with syndrome of inappropriate secretion of antidiuretic hormone (SIADH), nine were related to medication, and the remaining nine had other possible causes (adrenal insufficiency, loss through the gastrointestinal tract, malnutrition, incorrect measurement).

Among the 27 patients with SIADH, the cause was idiopathic in 16, whereas in 11 patients, pain, drugs, chronic obstructive pulmonary disease, and pneumonia were probable causes.

Among the 45 patients with hyponatremia, 37 had typical symptoms before the first consultation; lethargy 28/45, dizziness 20/45, unsteadiness and tendency to fall 17/45, and nausea 11/45. Headache was reported by 8/45, decreased appetite by 6/45 and concentration and memory difficulties by 3/45. At the time of referral, patients with hyponatremia had a median serum sodium level of 130 (IQR 128–132) mmol/L. Of these, 25 (56%) had a serum sodium level of 130 mmol/L or lower, and 4 (9%) had the same serum sodium level as the serum sodium level at the first consultation. At the first consultation, patients with hyponatremia had a median serum sodium level of 134 (IQR 130–137) mmol/L. Of these, 14 (31%) had a serum sodium level of 130 mmol/L or lower. At the last consultation, the level of serum sodium in all patients was over 130 mmol/L with a median of 137 (IQR 135–139) mmol/L (*P* < 0.001). The reference range of serum sodium at our laboratory was 137–145 mmol/L. Several patients received more than one measure, such as discontinuation of medication and then fluid restriction (Table 3). They were investigated and treated within a median time of 2 months with two consultations. Among patients with hyponatremia due to SIADH, 16/27 patients received fluid restriction down to 1000 ml/day as the first treatment option. Of these 16 patients, nine (56%) were able to implement the fluid restriction with subsequent normalization of their serum sodium. Those who did not carry out the fluid restriction, and who were asymptomatic with a serum sodium level above 130 mmol/L, did not receive any other treatment. Conversely, those who failed to carry out the fluid restriction but who were still symptomatic were treated with tolvaptan (vasopressin antagonist). Three patients received tolvaptan and they obtained normalization of serum sodium, became asymptomatic and reported no adverse reactions.

**Table 3:** Treatment measures (*N* = 60)

Type of measure^a^	Proportion	Comments
Discontinuation of drug	9/60	Mainly thiazide diuretics, ACE inhibitors, AII receptor antagonists, or combination preparations.
New medication/change of dosage	14/60	Mainly calcium channel blockers that replaced thiazide diuretics, ACE inhibitors, AII receptor antagonists or a combination preparation. Three patients received tolvaptan.
Fluid restriction	16/60	Maximum fluid restriction was down to 1000 ml per day.
Advice and information	20/60	About what the cause of the electrolyte imbalance was and how this could be prevented as well as dietary advice.
No measures	1/60	Hyponatremia was most likely a measurement error.

^a^ Last measure that was implemented

### Admissions

**Fig. 1:**
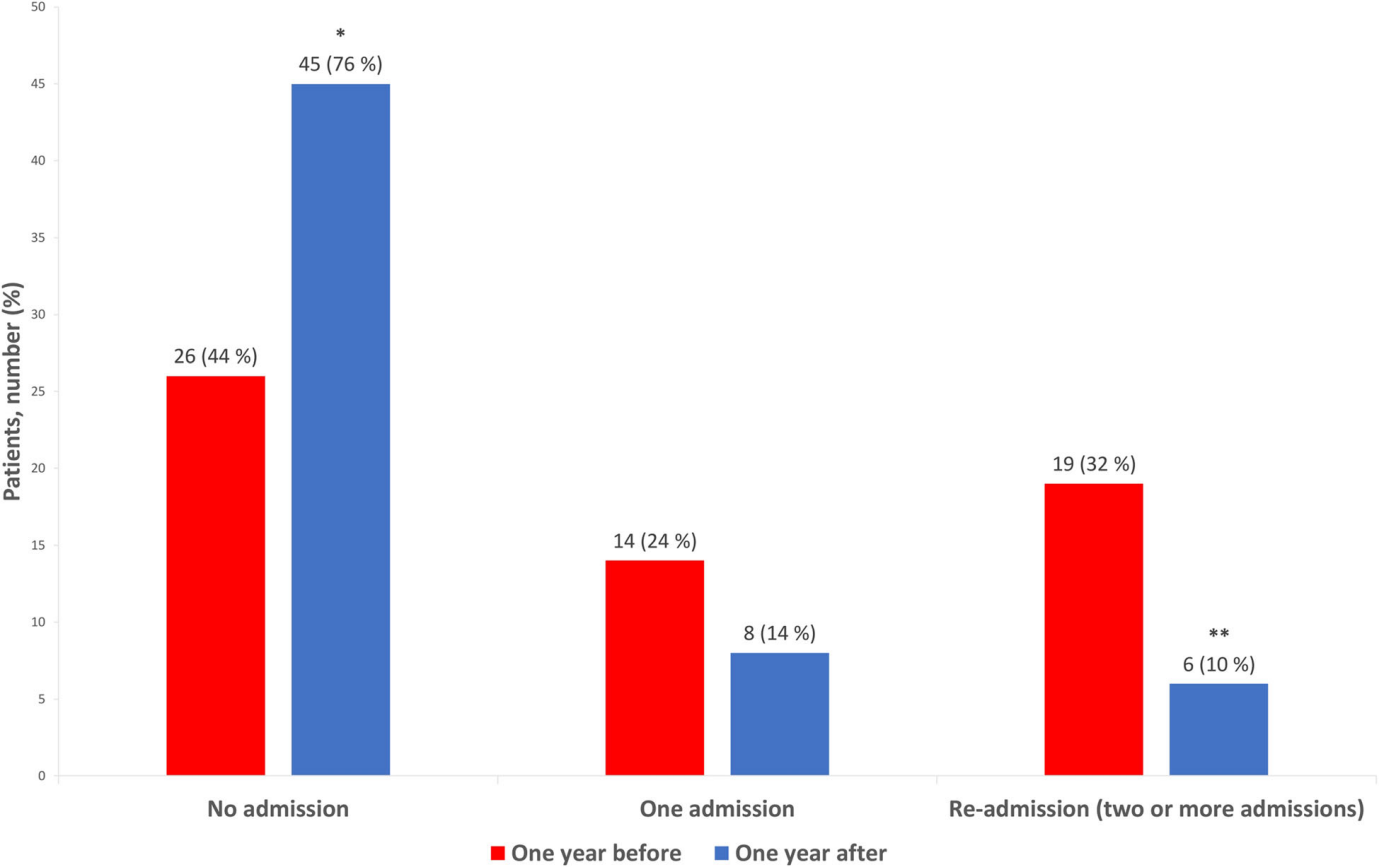
Number of patients admitted with the same electrolyte imbalance 1 year before the first consultation and 1 year after the last consultation at the electrolyte outpatient clinic, *N* = 59 * *P* < 0.001; ** *P* 0.003

All patients who were alive (59/60) 1 year after the last consultation had a total of 71 admissions with the same ED during the year before the first consultation, of which 66 admissions were due to hyponatremia. There were 20 admissions with the same ED during the year following the last consultation; a reduction of 51 (72%). Nineteen patients were readmitted (two or more admissions) with the same EI during the year before the first consultation compared with six patients the year after the last consultation at the EOC (Fig. 1).

### Patient-reported outcomes via questionnaire

Of the 60 patients, 36 (60%) reported improvement in symptoms, while 24 (40%) reported unchanged symptoms. Thirty-seven 37 (62%) patients reported improved QoL, while 23 (38%) stated that their QoL was unchanged after the last consultation. Everyone, except one patient who did not answer, stated that they were satisfied with the service and how the EOC was organized.

## Discussion

Most patients with EI had hyponatremia and were referred to the EOC by internists and general practitioners. Almost all had comorbidities and were older. The number of admissions with the same EI decreased after treatment at the EOC, although due to limitations in the study design it cannot be concluded that the EOC caused the reduction in admissions. Most patients (60%) reported an improvement in symptoms and 62% reported an improvement in QoL after treatment at the EOC. This improvement occurred in parallel with normalization of electrolyte values, so it is likely that the treatment offered at the EOC contributed to the improvement of symptoms and QoL. Nevertheless, other reasons for this improvement cannot be ruled out due to the limitations of our study.

### Hyponatremia

Most of our patients had hyponatremia, which is known as the most common EI [7]. The main reason for hyponatremia in our patients was idiopathic SIADH. SIADH is a diagnosis of exclusion, and several criteria must be met for this diagnosis [16]. Earlier studies have shown that idiopathic SIADH occurs in up to 60% of elderly patients with hyponatremia [17–19], which corresponds to our findings. The first choice of treatment of idiopathic SIADH is fluid restriction [16] and 56% of our patients with fluid restriction obtained normal serum sodium compared with 68% in another study [17]. At the first consultation 14/45 patients with hyponatremia had a serum sodium level of 130 mmol/L or lower. A meta-analysis has shown that correction of hyponatremia is associated with a reduced risk of mortality of up to 70% for a correction of serum sodium levels to above 130 mmol/L [20]. Readmissions with hyponatremia are common in older patients and are associated with higher mortality compared with patients who are admitted only once with hyponatremia [10]. After the patients were investigated and treated at the EOC, the number of admissions with the same EI decreased. Our experience supports the importance of investigating and treating patients with hyponatremia, especially those with a chronic disturbance.

### Strengths and limitations

We do not know similar outpatient clinics for EI, nor have we found publications describing similar services. All patients were investigated, treated and followed up by one physician (KT), which is an advantage and strength, but a limitation when it comes to assessing whether this model is applicable in other hospital settings. It is a strength that our study included patient-reported outcomes like symptoms and QoL, as well as admissions, unlike many other studies that have studied the association between EI and morbidity, mortality, and hospital costs only. However, there are limitations to our study which was designed as an uncontrolled before-and-after study. When designing the study it was not practical to have a control group, since the patients assigned to the control group would most probably be seen by a physician at the medical outpatient clinic, who would consult KT (the physician who managed the EOC) about the diagnostic workup and treatment. Therefore, we decided to investigate each individual patient as their own control. Consequently, we cannot conclude that the EOC itself reduced the rate of admissions or lead to improvement of symptoms of the patients. However, it is possible that the lower readmission rate, and the improvement in symptoms could be the result of the intervention. Another limitation is the questionnaire that was self-composed and not validated or tested for reliability. However, validated questionnaires suited to our study population were not possible to find. We only received 90 referrals during the five-year period, and we think there were two main reasons for this. Firstly, the physician at the EOC received many requests regarding patients admitted to the hospital, which were handled while they were in-patients. In addition, the physician at the EOC had many telephone conferences with hospital doctors and general practitioners which reduced the need for referrals. Secondly, despite providing information about the outpatient clinic twice a year to the hospital doctors via email and to general practitioners via letter of information, we still think that some doctors were not familiar with this service. The low number of referrals indicates that it is most appropriate to organize an EOC as a flexible service with well-defined investigation packages in a medical outpatient clinic and not as an independent outpatient clinic. Since the patients were mainly older and with comorbidities the structured investigation of EI, such as described here, could be organized in an outpatient clinic for endocrinology, nephrology, or geriatrics. However, the most important is probably not the affiliation of the service, but physicians with knowledge and experience about EI in addition to well-defined investigation programs.

## Conclusion

Our experience from the EOC is that it may be appropriate for patients with EI who are not adequately taken care of in other parts of the healthcare system. Our findings and experiences indicate that such an outpatient service can improve diagnostics and treatment, with the improvement of patient-reported outcomes. Since symptoms of hyponatremia are often nonspecific, it is important to investigate such symptoms and not simply think they are due to old age or comorbidity.

Due to the limitations of our study, our findings and the effectiveness of an EOC should be confirmed by a randomized controlled clinical trial.

## Supplementary information

**Additional file 1.** Questionnaire for electrolyte outpatient clinic

## Data Availability

The datasets used and/or analyzed during the current study are available from the corresponding author on reasonable request.

## Abbreviations

EI: Electrolyte imbalance
EOC: Electrolyte outpatient clinic
IQR: Interquartile range (IQR)
QoL: Quality of life
SIADH: Syndrome of inappropriate secretion of antidiuretic hormone
