# Supplementary material for: Electrolyte outpatient clinic at a local hospital – experience from diagnostics, treatment and follow-up
Source: BMC Health Serv Res. 2020 Feb 28;20:154. doi: 10.1186/s12913-020-5022-0 (PMC7048094; doi:10.1186/s12913-020-5022-0)
Supplement: Supplementary file 1 — Additional file 1. Questionnaire for electrolyte outpatient clinic [file 12913_2020_5022_MOESM1_ESM.pdf]

Questionnaire for electrolyte outpatient clinic

Diakonhjemmet Hospital

1. **Gender**

☐ Male ☐ Female

2. **Age**

☐ 20-29 ☐ 30-39 ☐ 40-49 ☐ 50-59 ☐ 60-69 ☐ 70-79 ☐ 80-89 ☐ 90-99

3. **Was the notice letter to the outpatient clinic informative enough?**

☐ Yes ☐ No ☐ Don't recall

Comments:

4. **Was it OK to have blood tests done and hand in a urine sample on the previous or same day as the consultation?**

☐ Yes ☐ No

Comments:

5. **Did you have any difficulties finding the electrolyte outpatient clinic?**

☐ Yes ☐ No

Comments:

Diakonhjemmet Hospital

6. How long did you wait in the waiting room before seeing the doctor?

☐ < 10 minutes ☐ 10-15 minutes ☐ 16-30 minutes ☐ > 30 minutes

7. Did you have sufficient time with the doctor?

☐ Yes ☐ No

Comments:

8. Did you get enough information/explanation from the doctor?

☐ Yes ☐ No

Comments:

9. Did you have any symptoms before going to the electrolyte outpatient clinic?

☐ Yes ☐ No

If yes, please specify in the comment box below.

Comments:

10. Did you notice any change in your symptoms after you attended the outpatient clinic?

☐ Improvement of symptoms ☐ No change of symptoms ☐ Worsening of symptoms

If you experienced an improvement or deterioration in your symptoms, please specify in the comment box below.

Comments:

11. Did you notice any change in quality of life after attending the electrolyte outpatient clinic?

☐ Improved quality of life ☐ no change in quality of life ☐ Worsened quality of life

Please specify in the comment box below.

Comments:

12. What did you think of the electrolyte outpatient clinic?

☐ Very bad ☐ Bad ☐ OK ☐ Good ☐ Very good

Comments:

13. Would you recommend the electrolyte outpatient clinic to others?

☐ Yes ☐ No

Comments:

14. Do you have any suggestions for improving the electrolyte outpatient clinic?

☐ Yes ☐ No

If yes, please specify in the comment box below.

Comments:

Thank you very much for taking the time to complete this questionnaire.
